# Nitrogen Doped Carbons Derived From Graphene Aerogel Templated Triazine-Based Conjugated Microporous Polymers for High-Performance Supercapacitors

**DOI:** 10.3389/fchem.2019.00142

**Published:** 2019-04-17

**Authors:** Lan Peng, Qianyin Guo, Zhaolin Ai, Yan Zhao, Yunqi Liu, Dacheng Wei

**Affiliations:** ^1^State Key Laboratory of Molecular Engineering of Polymers, Department of Macromolecular Science, Fudan University, Shanghai, China; ^2^Institute of Molecular Materials and Devices, Fudan University, Shanghai, China; ^3^Department of Material Sciences, Fudan University, Shanghai, China

**Keywords:** conjugated microporous polymer, graphene aerogel, supercapacitors, triazine-based electrode materials, nitrogen-doped carbon

## Abstract

Conjugated microporous polymers (CMPs) have attracted intensive attention owing to their permanent nanoporosity, large surface area and possibility for functionalization, however their application in energy storage suffers from poor conductivity and low hetero-atom content. Here, we demonstrate a hybrid of conjugated microporous polymers and graphene aerogel with improved conductivity. After treating at 800°C in NH_3_, the nitrogen content increases to 9.8%. The resulting microporous carbon exhibits a significant rise in supercapacitive performance up to 325 F g^−1^, 55% higher than pristine triazine-based CMPs, with energy density up to 12.95 Wh kg^−1^. Moreover, it has high stability with 99% retention after 10,000 cycles at 5 A g^−1^. The synergy of hierarchical porous structure, graphene-based conduction path and high percentage of hybridization with nitrogen ensures effective ion/electron transport and diffusion, making NH_3_-treated graphene aerogel/CMP hybrid a promising electrode material in high-performance supercapacitor.

## Introduction

Conjugated microporous polymers (CMPs) are one kind of covalently linked organic porous materials which have attracted extensive interest in recent years due to their strong π-conjugated linkage, permanent nanoporosity, large surface area, possibility to modify functional groups as well as high stability compared with other porous organic materials (Xu et al., 2013). Until now, CMPs have been used in various fields, including gas adsorption and storage (Yuan et al., 2010; Reich et al., 2012), gas separation, heterogeneous catalysis, light harvesting devices, photoluminescence, electric energy conversion and storage, etc. Among them, supercapacitor, as one kind of electrochemical energy storage devices, shows great potential for daily appliances (Pang et al., 2015; Zhang et al., 2015, 2016; Xie and Zhang, 2016) owing to its fast charge-discharge rate, high power density, less environment pollution, etc. (Zhang and Zhao, 2009; Wang G. et al., 2012; Peng et al., 2014; Salunkhe et al., 2014; Wang Q. et al., 2014)Compared with traditional supercapacitor electrode materials, CMPs have high specific surface area and the possibility to tailor pore and channel structures. Recently, the CMPs are normally produced by solvothermal method, which is suitable for carbon-carbon bond-forming reactions. However, CMPs produced by solvothermal method have relatively poor orbital overlap due to the twisted benzene rings, leading to low electrical conductivity (Lee et al., 2016). To improve the conductivity, CMPs with high carbon content are synthesized by coupling reactions, however it usually results in poor wettability of CMPs-based electrode.

To improve the performance of CMPs as electrode material, hybridization with other heteroatoms are widely used, which can increase both the wettability and the pseudocapacitance. With higher nitrogen content, the supercapacitive performance of a triazatruxene-functional CMP are remarkably improved, however the value only reaches 183 F g^−1^ (Li et al., 2017). Moreover, to increase the material conductivity, CMPs are usually carbonized under high temperature, which graphitizes the framework of carbon-based materials (Li et al., 2014; Wang L. et al., 2014). As an example, Cooper et al. improve the electrical conductivity and pseudocapacitive contributions of CMPs by combining molecular design and carbonization under an ammonia atmosphere, however the value only increased to about 260 F g^−1^ and the performance still restricts by the limited intrinsic conductivity of CMPs, which is also a common problem for other porous organic electrode materials (Lee et al., 2016).

Here, graphene aerogels (GA) are hybridized with CMPs, for the first time, to improve three-dimensional intrinsic conductivity of CMPs. After carbonization under ammonia atmosphere, the nitrogen content increases to 9.8%, while the ratio of pyridinic nitrogen increase from 15 to 25.5%. Owing to the improved electrical conductivity and higher nitrogen content, the resulting NH_3_-treated GA/CMPs (N-GA /CMPs) has extraordinary specific capacitance up to 325 F g^−1^ at 0.5 A g^−1^ with excellent long-term cycling stability, showing its great potential in high performance supercapacitors.

## Materials and Methods

### Preparation of CMP, GA/CMP, N-GA/CMP

CMPs were synthesized by the ionothermal method (Kuhn et al., 2008a). The proportional (1:20) monomer (m-phthalodinitrile) and ZnCl_2_ were added to a Pyrex ampoule in an argon atmosphere, and then the ampoule was evacuated and sealed. With a 10°C min^−1^ heating rate, the ampoule was heated to 600°C for 40 h, and then was cooled down to room temperature. If the synthesis temperature was higher than 500°C, the ampule was under pressure and would be released during opening (Kuhn et al., 2008b). The product was subsequently grounded in an agate mortar to get powder, and washed by deionized water to remove ZnCl_2_. After that, the sample was stirred in 5% HCl for 15 h to remove the residual ZnCl_2_. After purification, the black powder was filtered and washed with deionized water and tetrahydrofuran. Finally, it was dried in vacuum at 150°C for 15 h. Graphene oxide was synthesized from natural graphite according to a modified Hummers method (Hummers Jr and Offeman, 1958). The prepared graphene oxide solution was purified by 5% HCl and deionized water for several times so that residual salts and acids can be washed completed. Graphene Aerogel was synthesized according to the literature (Xu et al., 2010). A 10 mL portion of 1.5 mg mL^−1^ homogeneous graphene oxide aqueous dispersion was sealed in a 20 mL Teflon-lined autoclave and maintained at 180°C for 12 h. The autoclave was naturally cooled to room temperature, and the as-prepared graphene aerogels were taken out and transferred to glass bottles. The graphene aerogels were obtained by putting the glass bottles into a freeze drying equipment for 24 h. The preparation of GA/CMPs followed the preparation of CMPs but different ratio of graphene aerogels, monomers and ZnCl_2_ were grinded evenly in an agate mortar before adding to a Pyrex ampoule. The weight percentage of GA was 15%. To prepare N-GA/CMP, GA/CMPs (200 mg) was placed in a ceramic boat, which was located in the center of a tube furnace. The sample was exposed to a flow of N_2_ for 30 min to remove the air from the tube, and then was treated by ammonia at 800°C for 2 h using nitrogen as the carrier gas. After ammonia treatment, the sample was cooled to room temperature and NH_3_-treated GA/CMP was obtained.

### Material Characterization

The samples were measured by scanning electron microscopy (SEM, Zeiss-Ultra 55), transmission electron microscopy (TEM, Tecnai G2F20S-Twin), X-ray diffraction (XRD, Rigaku D/Max 2400, CuKα radiation, 40 kV, 100 mA, λ = 1.5406 Å), and X-ray photoelectron spectroscopy (XPS, Axis Ultra Dld, Al Kα radiation, 15 kV, 30 mA). Elemental analyses were carried out using a vario EL cube analyzer. The pore structure was measured by N_2_ sorption at 77 K, Brunauer–Emmett–Teller (BET) surface areas were calculated from the isotherm using the BET equation.

### Fabrication of Electrodes and Supercapacitor

The active material (80 wt.%), carbon black (10 wt.%), and polytetrafluoroethylene (PTFE, 10 wt.% of a 60 wt.% dispersion in water) were dispersed in ethanol, and then thoroughly mixed in an agate mortar, adding ethanol several times. After that, the mixtures were rolled into a uniform thin film and dried at 80°C for 12 h in a vacuum oven, and then film was cut into 1 cm^2^ electrode slice and coated on a slice of nickel foam current collector. Exerted 10 MPa pressure by a table press, the as-prepared electrodes were used as the working electrode. Pt foil and an Hg/HgO electrode were used as the counter electrode and reference electrode, respectively.

### Electrochemical Measurement

Electrochemical measurement was performed by using an electrochemical station (CHI660E, CH Instruments, Shanghai) and a three-electrode system in 6 M KOH alkaline electrolyte. The specific capacitances of materials derived from galvanostatic discharge curves were calculated by Equation (1):

(1) $$\begin{array}{r} C_{m}=\frac{I_{d}\times \Delta t}{m\times \Delta V} \end{array}$$

where Δ*t* was the discharge time (s), *I*_d_ was the constant discharge current (mA), Δ*V* was the potential change (excluding ohmic drop IR), *m* was the mass of active materials in the working electrode when using three-electrode system. In two-electrode system, *m* was the mass of active material of whole device (mg).

The energy density (*E*, W h kg^−1^) and average power density (*P*, W kg^−1^) of the whole two-electrode device were calculated separately by Equations (2, 3):

(2) $$\begin{array}{r} E=\frac{1}{2}\times \frac{C_{m}\times \Delta V^{2}}{m\times 3.6} \end{array}$$

(3) $$\begin{array}{r} P=3600\times \frac{E}{\Delta t} \end{array}$$

## Results and discussion

**Figure 1:**
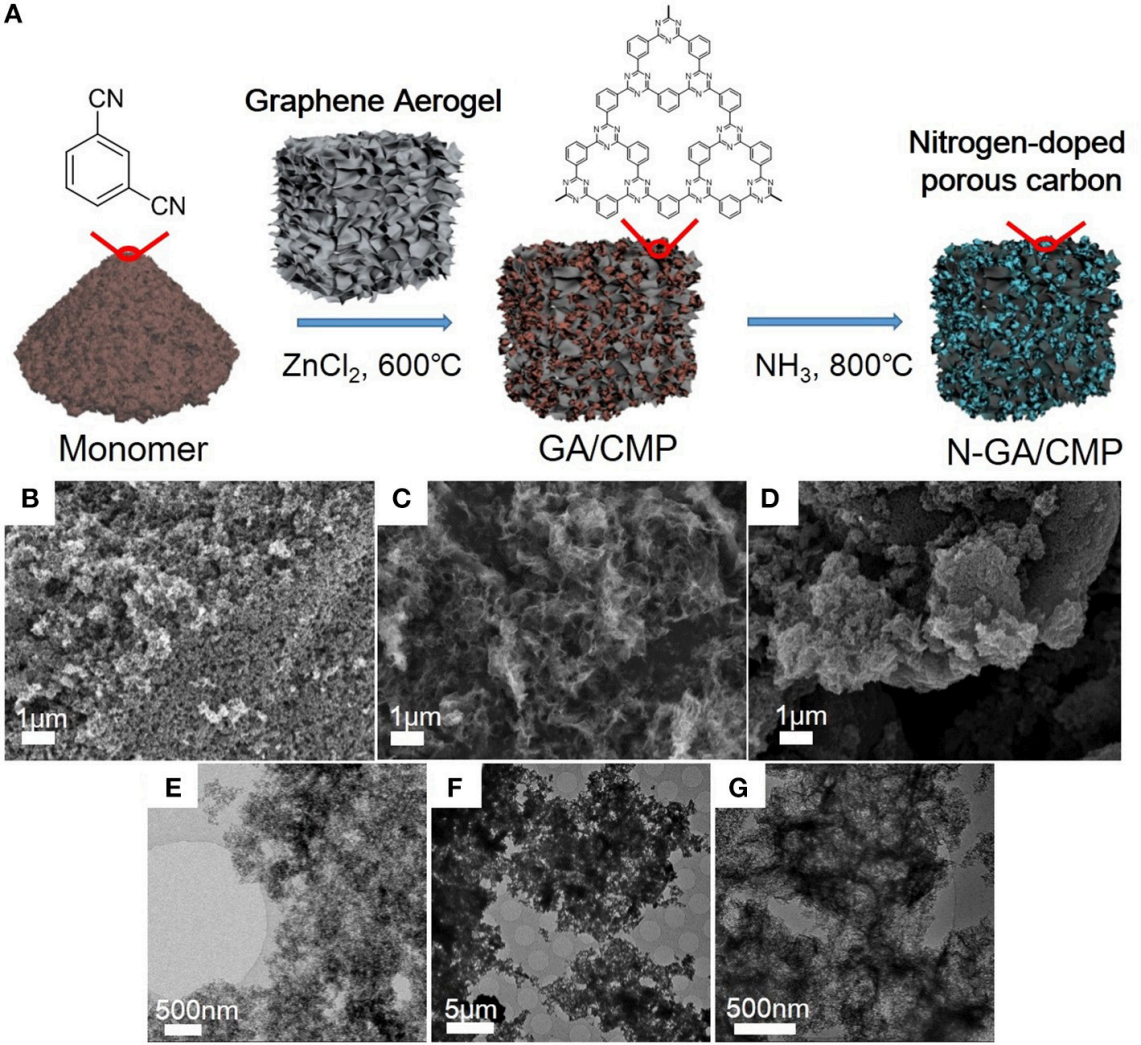
**(A)** Illustration of synthesis of N-GA/CMPs. **(B–G)** FESEM and TEM images of the CMPs **(B,E)**, GA **(C,F)**, and N-GA/CMPs **(D,G)**, respectively.

**Figure 2:**
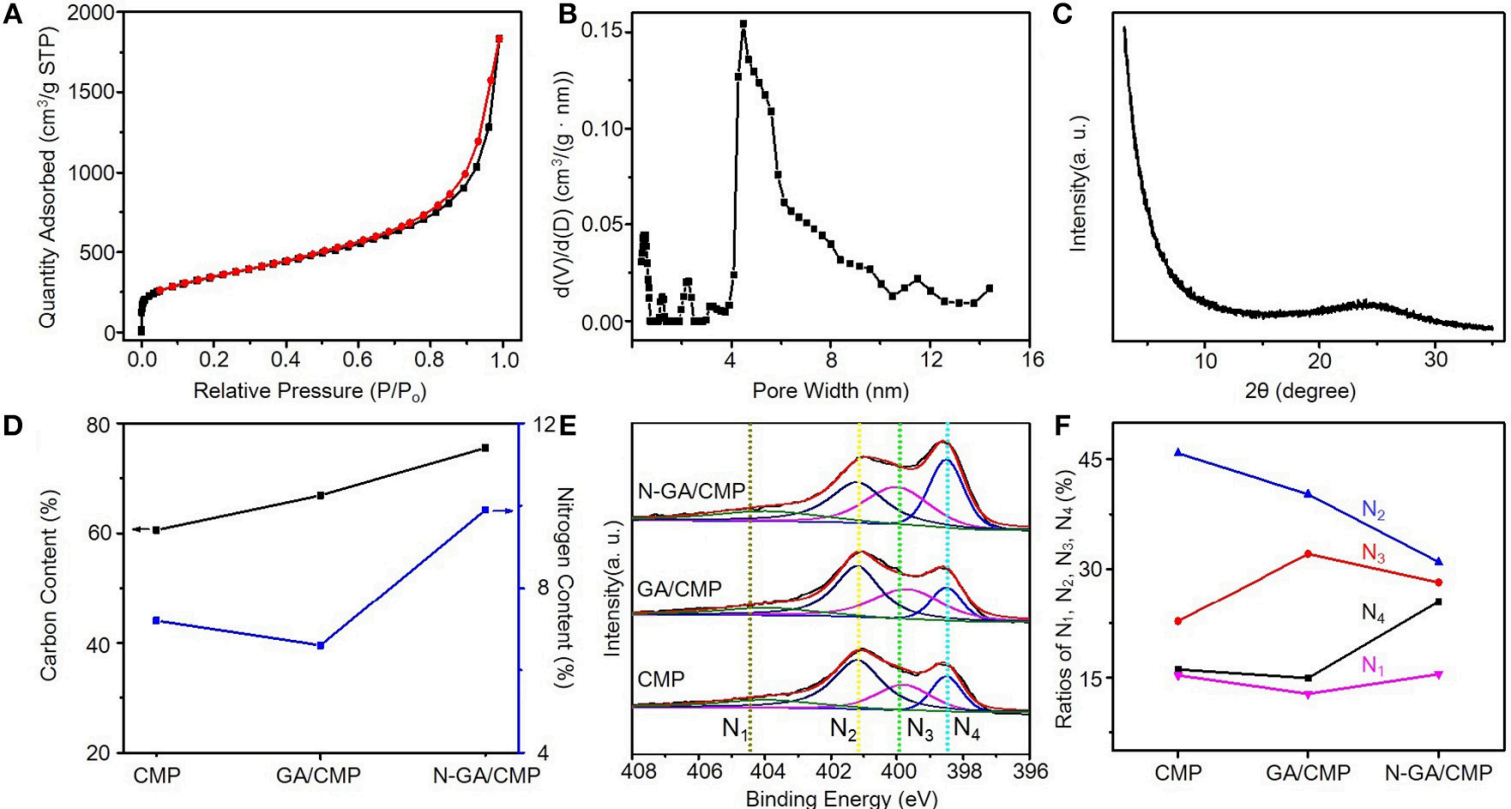
**(A)** Nitrogen adsorption and desorption isotherms of CMPs (red) and N-GA/CMPs (blue). **(B)** Pore size distribution of CMPs and N-GA/CMPs. **(C)** XRD pattern of N-GA/CMPs. **(D)** EA of N-GA/CMPs for carbon (black) and nitrogen (blue). **(E)** XPS N 1s spectra of CMPs, GA/CMPs and N-GA/CMPs (N_1_, oxide nitrogen; N_2_, quaternary nitrogen; N_3_, pyrrolic nitrogen; N_4_, pyridinic nitrogen). **(F)** Ratios of different configurations of nitrogen in the materials.

After growth (Figure 1A), the morphologies of CMPs, GA, and N-GA/CMPs were studied by field emission SEM (FESEM, Figures 1B–D) and TEM (Figures 1E–G). The FESEM image shows that the CMPs have similar porous structures with the triazine-based CMPs reported in literature (Kuhn et al., 2008b). The TEM image of the N-GA/CMPs shows that the GA skeleton with layered structure is cross-linked and uniformly covered by porous CMPs. Nitrogen adsorption-desorption measurement (Figures 2A,B) has been performed to analyze the porous structure of the N-GA/CMPs. The isotherm of the N-GA/CMPs reveals a typical II type, which indicates the combination of micro-pores and meso-pores in the material. The surface area of N-GA/CMPs measured by BET is 1268.5 m^2^ g^−1^, and the powder XRD pattern shows that the N-GA/CMPs don't have any crystallization peaks, proving the amorphous nature (Figure 2C).

To further study the chemical structure and elemental composition, N-GA/CMPs were characterized by elemental analysis and XPS. The elemental analysis shows that the pristine GA/CMPs have a 6.3% nitrogen content (Figure 2D) (Hao et al., 2014). After carbonization at 800°C in ammonia, the nitrogen content of N-GA/CMPs increases to 9.8%. In order to analyze the nitrogen species in the material, we measured XPS spectra of CMPs, GA/CMPs and N-GA/CMP (Figure 2E). The XPS N 1s has four sub-peaks at 398.5, 399.8, 400.9, and 403.1 eV, which represent pyridinic nitrogen, pyrrolic nitrogen, quaternary nitrogen and oxidized nitrogen, respectively. Except quaternary nitrogen, other three types of nitrogen are located at the edges or defects of the CMPs or GA layers. All examples have low percentage of oxidized nitrogen, since the reactions were token place under an inert atmosphere. Notably, after treating at high temperature in ammonia atmosphere, no obvious changes happen on pyrrolic and oxidized nitrogen, while the ratio of quaternary nitrogen decreases and the ratio of pyridinic nitrogen remarkably increases up to 25.5% (Figure 2F), which is of importance for achieving high supercapacitive performance.

**Figure 3:**
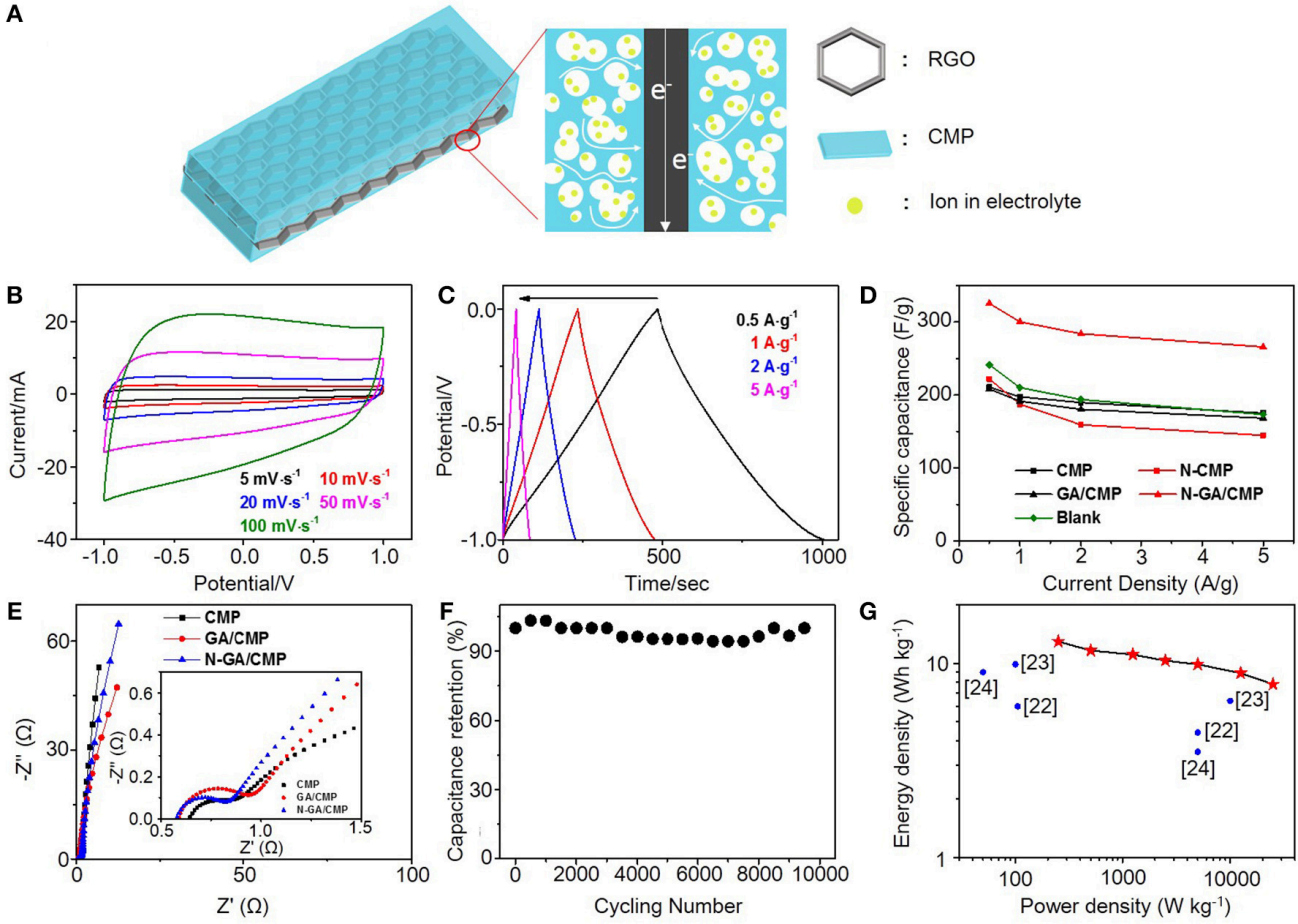
**(A)** Illustration of the charge transfer in an N-GA/CMPs electrode. **(B)** CV curves and **(C)** GCD curves of the N-GA/CMPs. **(D)** Specific capacitances of CMPs, N-CMPs, GA/CMPs, N-GA/CMPs, GA and physical mixture of 85% CMP and 15% GA as blank. **(E)** Nyquist plots of the CMPs, GA/CMPs and N-GA/CMPs. The insert shows the plots in the high-frequency region. **(F)** Cycling performance of the N-GA/CMPs based supercapacitor. **(G)** Ragone plots of the N-GA/CMPs based two-electrode supercapacitor. The values reported by literatures are shown in the figure.

To evaluate the supercapacitive performance, cyclic voltammetry (CV), galvanostatic charge–discharge (GCD) tests and electrochemical impedance spectroscopy (EIS) were carried out in a 6M KOH aqueous electrolyte in a conventional three-electrode system (Figure 3A). The CV curves for N-GA/CMPs at different scanning rates are shown in Figure 3B. All CV curves have quasi-rectangular shape without obvious reduction and oxidation peaks, indicating the N-GA/CMPs have good electroconductivity and stores energy mainly by double-layer energy storage mechanism despise higher content of pyridinic nitrogen. The GCD tests are performed at different current densities from 0.5 to 5 A g^−1^. The GCD curves (Figure 3C) have a triangular shape, consistent with theoretical electrochemical double layer capacitors, and don't have obvious voltage drop, showing the good conductivity of materials. The N-GA/CMPs exhibit the highest specific capacitance of 325 F g^−1^ at the current density of 0.5 A g^−1^. The calculated specific capacitance values of CMP, GA/CMP and N-GA/CMP (Figure 3D) show that the GA cannot greatly increase the specific capacitance, while the high-temperature annealing in ammonia atmosphere leads to a great enhancement. To understand the synergy effect of the GA and the CMPs in the hybrids, we physically mixed same content GA with CMPs and annealed at the same temperature in ammonia atmosphere (named as blank group). The specific capacitance of the physical mixture is nearly same with the CMPs (Figure 3D), implying that the increase of specific capacitance mainly results from the chemical combination of porous structure of CMPs and in-plane conductive structure of GA (Figure 3A). The CMPs cover on both sides of GA. The interconnected micro-pores and meso-pores of CMPs shorten the diffusion length between external electrolyte and the surface of electrode materials. At the same time, the GA acts as a conductive framework to increase the conductivity of the electrodes.

The Nuquist plot (Figure 3E) measured by EIS reveals the device resistance including the electrolyte resistance, the ionic resistance of ions moving through the separator, the intrinsic resistance of the active electrode, and the contact resistance at the interface between the active materials with the current collector. In the high-frequency region, the equivalent series resistance (ESR) of the N-GA/CMPs (0.58 Ω) is smaller than that of CMPs (0.65 Ω) after N-doping, which indicates lower charge transfer resistance at the electrode/electrolyte interface. The smaller radius of semicircular and the more vertical line in the low-frequency region indicate faster ion diffusion in the electrode of the N-GA/CMPs. This phenomenon is attributed to higher ratio of nitrogen in the N-GA/CMPs (Xiang et al., 2015), in agreement with the EA.

In order to test the stability in the application, the N-GA/CMPs were used in a conventional two-electrode system for cycling experiments. The capacitance (Figure 3F) remains above 95% after 10,000 charge-discharge cycles at a current density of 5 A g^−1^. Therefore, the N-GA/CMPs produced by ionothermal synthesis possess not only high supercapacitive performance but also excellent cycling life. The energy density of the N-GA/CMPs supercapacitors (Figure 3G) is up to 12.95 Wh kg^−1^, which is among the highest reported value of the CMP-based supercapacitors (Xu et al., 2017; Yuan et al., 2017;Zhao et al., 2017).

## Conclusions

In summary, GA templated triazine-based CMPs are synthesized by ionothermal method followed by high temperature treatment in ammonia atmosphere. By using GA as the template, the inherent conductivity of triazine-based CMPs is improved. High temperature process in ammonia atmosphere results in higher pyridinic nitrogen content in the GA/CMPs, which introduces more edge sites and defects in the material. As a result, the specific capacitance of N-GA/CMPs electrode increases by 55% up to 325 F g^−1^, and the energy density reaches 12.95 Wh kg^−1^. Moreover, there is no significant degradation after 10,000 cycles at a current density of 5A g^−1^. Considering the easy preparation and outstanding energy storage performance, the N-GA/CMPs show great potential for practical application in high-performance aqueous supercapacitors.

## Author Contributions

DW designed research. DW, YZ, and YL supervised the project. LP, QG, and ZA performed the experiments. DW and LP wrote the manuscript. All authors commented on the manuscript.

### Conflict of Interest Statement

The authors declare that the research was conducted in the absence of any commercial or financial relationships that could be construed as a potential conflict of interest.
